# Magnetic Resonance Imaging in Acute Ischemic Stroke

**DOI:** 10.7759/cureus.27224

**Published:** 2022-07-25

**Authors:** Eric Hartono Tedyanto, Kumara Tini, Nyoman Angga Krishna Pramana

**Affiliations:** 1 Department of Neurology, Universitas Udayana/Sanglah General Hospital, Denpasar, IDN

**Keywords:** aspect score, aspect, thrombectomy, thrombolysis, mri, ischemic stroke

## Abstract

Ischemic stroke is one of the leading causes of mortality and disability. The only effective non-surgical treatment for acute ischemic stroke within three to four and a half hours of the onset of symptoms is thrombolytic therapy. Time is of the essence when diagnosing and treating an acute ischemic stroke. After evaluating the advantages and disadvantages of thrombolysis, selecting the ideal patient for the indication is essential. Magnetic Resonance Imaging (MRI) is more sensitive and specific than Computed Tomography (CT) scans when identifying acute ischemic stroke. In approximately 80% of cases, infarcts are detectable within the first 24 hours. MRI can detect an ischemic stroke within a few hours of its onset. Multimodal imaging provides information for the diagnosis of ischemic stroke, patient selection for thrombolytic therapy, and prognosis estimation.

## Introduction and background

Ischemic stroke is one of the leading causes of death and disability. In recent years, several attempts have been made to improve the efficacy of acute ischemic stroke therapies. Thrombolytic therapy is the only effective treatment for acute ischemic stroke within three to four and a half hours after the beginning of symptoms. Ongoing research focuses on thrombolysis indications and therapeutic window treatment. After assessing the risks and benefits of thrombolysis, selecting a suitable patient for the indication is essential [1,2]. A CT scan without contrast is a fast and reliable method for detecting cerebral bleeding, but it is not sensitive enough to detect ischemic changes in the early hours. Its sensitivity ranges between 40 and 60 percent in the first six hours following activation. The CT scan imaging is acquired prior to diffusion-weighted imaging (DWI), one of the high-resolution MRI sequences in regions with decreased blood flow [3].

“Time is brain” is essential for diagnosing and treating acute ischemic stroke. Every minute, the occlusion of the middle cerebral artery loses 9 million neurons. Brain imaging is the primary tool for diagnosing an ischemic stroke. Brain imaging helps differentiate between subacute and chronic lesions of the brain. This is essential for the selection of patients for thrombolysis or thrombectomy. In recent years, acute ischemic stroke assessment has dominated imaging investigations [4].

MRI is more sensitive and specific than a CT scan for diagnosing acute ischemic stroke. Study results show immediate non-contrast MRI is about five times more sensitive than and twice as accurate as immediate non-contrast CT for diagnosing ischemic stroke. Non-contrast CT and MRI were equally effective in the diagnosis of acute intracranial hemorrhage. Non-contrast CT has been the standard in emergency stroke treatment, primarily to exclude hemorrhagic stroke, which cannot be treated with clot-busting therapies. In approximately 80% of cases, infarcts are detectable within the first 24 hours. MRI can detect ischemic stroke within the first few hours after onset. Multimodal MRI is useful for ischemic stroke diagnosis and acute phase therapy planning. Different imaging results from the MRI sequence such as Diffusion-Perfusion mismatch determine the mechanism of the stroke, which influences the prognosis and is essential for determining the optimal treatment. The lesion mismatch profile on MRI aids in evaluating the potential risks and benefits of thrombolysis by providing information on the age of the ischemic lesion or information regarding salvageable tissue [3,5].

## Review

Magnetic resonance imaging sequence

The two most used MRI sequences are T1- and T2-weighted imaging. The image's contrast and brightness are determined mainly by the network's T1 characteristics. Typically, the CSF is a good indicator of the difference between T1- and T2-weighted imaging. CSF appears dark on T1 vs white on T2 [6]. Fluid Attenuated Inversion Recovery (FLAIR) is the third frequently utilized sequence. The FLAIR sequence has exceptionally long Time to Echo (TE) and Repetition Time (TR) but is otherwise similar to the T2 image. The anomaly will therefore continue to shine while the CSF will usually darken. Due to FLAIR's extraordinary sensitivity to disease, it is simpler to distinguish between abnormalities and CSF [7]. T1 imaging can also be done after Gadolinium injection. Gadolinium is a non-toxic, paramagnetic contrast-enhancing substance. Gadolinium reduces T1 when injected during imaging to change signal strength. Gadolinium appears highly bright on T1-weighted images as a result. Gadolinium-enhanced images give information on how vascular a lesion is and damage to the blood-brain barrier, such as tumors, abscesses, and inflammation (such as herpes simplex encephalitis, multiple sclerosis, etc.) [3].

Diffusion-weighted imaging (DWI) is designed to detect randomly moving water protons. Water molecules circulate freely in the extracellular region, despite a significant restriction in the intracellular region. Diffusion, the spontaneous movement process, rapidly separates ischemic brain tissue. During ischemia, the sodium-potassium pump ceases to function, accumulating sodium in the cells. The osmotic gradient induces water movement from the extracellular to the intracellular region. DWI reveals a powerful signal when intracellular water transport is restricted. DWI is a highly accurate method for diagnosing acute ischemia. DWI and apparent diffusion coefficient (ADC) detect and quantify this phenomenon within minutes of vessel occlusion. Ischemia cells can be distinguished from healthy cells on DWI MRI, which reveals a brighter lesion appearance than normal brain tissue by differences in water content and water diffusion [3,8]. A pseudo-diffusion coefficient map (ADC) was used to help identify areas of ischemia, and areas that were light in diffusion and dark in ADC were consistent with acute infarction. Because some high signal regions, such as vasogenic edema, may appear very bright on DWI, an ADC map is necessary. This is because T2-based DWI and this "enlightenment" can produce specific bright signals; however, these high-signal regions not caused by acute infarction are easily distinguished on the ADC map, allowing for an accurate initial diagnosis [9].

Perfusion-weighted imaging (PWI) can measure capillary perfusion in the brain. MR technology can perform cerebral hemodynamic imaging, which may be advantageous in certain situations. Typically, MR perfusion is used to confirm that the stroke syndrome is not caused by mimicking stroke but rather by hemodynamic variables. Patients without visible DWI lesions have the highest risk of developing the disorder. PWI has the same ability to detect an ischemic penumbra as perfusion CT. The penumbra is the mismatch between DWI and PWI. Extending the therapeutic window for treatment and guiding future ischemic stroke therapy will be aided by precisely identifying this ischemic penumbra [6].

Magnetic resonance imaging in acute ischemic stroke

MRI is a diagnostic tool sensitive enough to detect abnormalities in brain tissue and its environs. MRI has greater sensitivity and specificity in diagnosing acute ischemic stroke than CT scan. Approximately 80% of infarcts are detected within 24 hours. MRI can detect ischemic stroke within the first few hours of onset. MRI can differentiate between brain tissue at risk for infarction and brain tissue that has been irreparably damaged. Lacunar infarcts and brainstem infarcts can be identified by MRI, whereas CT scans have difficulty due to the surrounding bone. The MRI examines the underlying pathology and is sensitive to small infarcts. Compared to CT scans, the use of MRI is still relatively uncommon at present. This is because MRI is not available in all medical facilities and the MRI examination is more time-consuming than CT Scan [10].

Patients with an ischemic stroke may arrive at the hospital at different times after the onset of the event. The availability of information about the time of the stroke can aid in subsequent medical treatment. Multiple MRI sequences can assist in identifying the onset of a stroke. Based on the onset, a stroke can be classified as early hyper-acute, late hyper-acute, acute, subacute, or chronic. On conventional MRI, stroke has a characteristic feature that varies with the infarct's age. The temporal progression of stroke is typically classified as follows: hyper-acute (0-6 hours), late hyper-acute (6-24 hours), acute (1-7 days), subacute (7-21 days), and chronic stroke (>21 days) [11].

Hyper-acute (0-6 Hours)

Diffusion-weighted imaging reveals a rise in DWI signal and a drop in ADC (Appearance Diffusion Coefficient) values within minutes of arterial occlusion. At this stage, the affected parenchyma appears normal in other sequences, even though alterations in inflow (occlusion on MRA) and thromboembolism may be detected (e.g. on susceptibility weighted imaging {SWI}). Loss of normal blood flow and a high signal on T2/FLAIR and T1 C+ can also be used to detect slow or stagnant blood flow (intravascular elevation). Within minutes of onset, DWI can detect ischemic changes. Reduced proton motion is detected through a reduction in the ADC. PWI describes a decrease in cerebral blood flow (CBF) and cerebral blood volume (CBV) and an increase in blood-brain mean transit time (MTT) in early cerebral ischemia. The correlation between abnormalities in diffusion and perfusion and the infarct area indicates irreversible neuronal death. Diffusion and perfusion abnormalities that do not correspond to a perfusion abnormality more significant than a diffusion abnormality may indicate a region of reversible ischemic penumbra. Patients with mismatches may be eligible for stroke treatment. Two clinical trials have been completed testing the hypothesis that patients with an ischemic penumbra detected by a Diffusion-Perfusion mismatch benefit from thrombolysis beyond the 3-hour treatment period [3,12].

Late Hyper-acute (>6 Hours)

Typically, after six hours, a signal with a high T2 value is detected, which is initially more visible on the FLAIR than on the conventional fast spin-echo T2 10. Over the next two days, these alterations continued to intensify. T1 hypotension manifests itself only after 16 hours and persists [3].

Acute (1-7 Days)

Throughout the first week, the infarcted parenchyma displayed a high DWI signal and a low ADC signal; however, the ADC values had begun to rise by the end of the first week. The infarcts remained hyperintense on T2 and FLAIR, with T2 signals progressively increasing over the first four days. Some intrinsically high cortical T1 signals may be detected as early as three days after infarction, even though T1 signals remain low. After day 5, the cortex typically exhibits contrast improvement at T1 C+ 10 (usually). Less frequent enhancement patterns include arterial enhancement, which occurs in approximately half of strokes and becomes visible after three days, and rare meningeal enhancement, typically observed between 2 and 6 days [3].

Subacute (7-21 Days)

The pseudo-normalization of ADC typically occurs between 10 and 15 days. As the ADC value increased, the infarcted tissue gradually became lighter than the normal parenchyma. In contrast, the DWI remained elevated due to a persistently high T2/FLAIR signal (T2 glow), except for bleeding (T2 blackout) and cystic encephalomalacia. T2 fogging is typically observed between 1 and 5 weeks, most frequently during the second week. Due to cortical laminar necrosis or pseudo-laminar necrosis, T1 exhibits hypo-intensity with a high intrinsic cortical T1 signal. Typically, cortical enhancement occurs during the subacute phase [3,4].

Chronic (>21 Days)

T1 signal remains low, with intrinsic T1 high in the cortex in the presence of cortical necrosis. High T2 signal cortical contrast enhancement usually persists for 2 to 4 months. If parenchymal enhancement persists for more than 12 weeks, the presence of an underlying lesion should be considered [3].

Magnetic resonance imaging for differentiating infarct core and penumbra

When a cerebral artery is blocked, brain tissue nuclei perish rapidly. These infarcts are surrounded by hypo-perfused regions of the brain that survive due to collateral blood flow. The ischemic penumbra is potentially salvageable tissue in this at-risk region. When a cerebral blood vessel is blocked, a complex series of pathophysiological events unfold in space and time, according to the penumbra theory [12]. First, the core region quickly develops an infarct (ischemic core). Even when nerve function has ceased, the periphery of the nucleus still displays a minimal blood flow supplied by collateral circulation (ischemic penumbra). During the acute phase of an ischemic stroke, the nerve function of some portions of the ischemic penumbra may recover when blood supply is restored and undergo dynamic changes. Perfusion-weighted images (PWI) can identify ischemic penumbral tissue, while DWI lesions depict ischemic nuclei. Therefore, regions with PWI-DWI mismatch (i.e., when the perfusion lesion is greater than the diffuse lesion) represent salvageable tissue requiring immediate treatment [13].

Penumbra

In simple terms, it has been hypothesized that DWI reflects infarcts with irreversible damage, whereas PWI reflects hypo-perfused regions in general. This volume difference, also known as the PWI-DWI mismatch, represents the MRI correlation of the ischemic penumbra. In contrast, a PWI-DWI mismatch occurs when there is no difference in the volume of PWI and DWI or even a negative difference (PWI DWI). PWI/DWI concordance was observed in patients who lacked penumbral tissue, either due to the normalization of hypo-perfusion or the completion of the infarct and loss of the penumbra. It is unknown which parameter provides the best estimate for explaining hypo-perfusion and distinguishing the infarct from the penumbra. Multiple authors concur that T-max and mean transit time (MTT) produce the best outcomes in current clinical practice [3,14].

PWI-DWI Mismatch

PWI and DWI MRI provide greater sensitivity and specificity of information. However, there are advantages to imaging with CT scans, including the fact that CT scans are more widely available than MRI, allowing imaging tests to be conducted earlier. MRI perfusion imaging is not commonly used. One of the primary benefits of perfusion-MR (PWI = perfusion-weighted imaging) is that it can evaluate the perfusion of the entire brain. Like CT perfusion, PWI MRI can detect ischemic penumbra, the difference between a DWI defect (irreversible cytotoxic-ischemic edema - ischemic core) and a perfusion-analogous defect with MTT or TTP (time-to-peak). The penumbra is a mismatch of DWI and PWI. Accurate identification of this ischemic penumbra will guide future therapy for ischemic stroke and may help prolong treatment duration [15,16].

DWI-FLAIR Mismatch

An MRI feature combining fluid attenuation inversion recovery (FLAIR) sequence with DWI has been studied as a determinant of lesion age, and a DWI-positive/FLAIR-negative mismatch pattern was identified in patients within 6 hours of stroke onset in the middle cerebral artery region, with high predictive value [4]. Clinicians are frequently faced with patients whose exact time of onset of stroke symptoms is unknown, and attempts have been made to use signal changes in FLAIR images as a sort of "tissue clock." It is known, for instance, that the signal intensity in FLAIR images increases proportionally to the increase in water content in infarcted tissue. Within 1 to 4 hours after the onset of a stroke, an increase in water content occurs due to vasogenic edema caused by a breach in the blood-brain barrier. DWI-FLAIR mismatch (i.e., lesions seen on DWI but not FLAIR) has therefore been used as a surrogate marker to estimate the age of the unknown stroke-onset lesion and may aid in determining the use of thrombolytic agents. Patients with DWI-FLAIR mismatch are typically within the thrombolysis time window, with high specificity and predictive value (93 percent and 94 percent ) [10]. In the RESTORE study, patients with unclear stroke onset, symptom detection with a PWI-DWI >20% mismatch within 6 hours, and negative or subtle FLAIR changes were treated with reperfusion therapy. MRI-based reperfusion therapy was found to be feasible and safe when combined with tissue plasminogen activator (t-PA) or endovascular therapy, and MRI-based reperfusion therapy was also found to be feasible [10,17].

However, potential confounding factors can also reduce the diagnostic accuracy of DWI-FLAIR mismatch. In addition to the duration since the onset of symptoms, a high FLAIR signal intensity has been associated with younger age and a greater number of ischemic lesions. As age and lesion size alter the permeability of the blood-brain barrier, these biological variations may cause alterations in the FLAIR signal intensity. In addition, there is a problem resulting from the DWI-FLAIR mismatch's low reading ability. Further information regarding the risks and benefits of MRI-based thrombolysis in this particular group of patients may be gleaned from ongoing clinical trials [10].

Comparing the signal intensity on DWI, ADC, and FLAIR images can assist in distinguishing between acute, subacute, and chronic stroke. A positive signal on DWI without a corresponding FLAIR hyperintensity indicates that the stroke occurred between 4.5 and 6 hours prior to imaging (62% sensitivity, 78% specificity). In the first 7 to 10 days, the ADC value decreases, then pseudo-normalizes (the period in stroke evaluation when the ADC is normal), and then increases. Given the temporal relationship between the MRI sequences, concurrent viewing of the MRI sequences can assist in identifying patients with "unknown onset" or wake-up stroke of uncertain onset [9,10,18].

Alberta stroke program early CT (ASPECT) score and intravenous thrombolysis

An ASPECT score greater than 7 may be a good predictor of functional outcome and bleeding transformation in ischemic stroke treated with alteplase. The ASPECTS scoring system is the foundation for intravenous thrombolysis treatment decisions [19]. However, developments and additional research appear to be causing a shift in perception. According to the 2018 American Heart Association/American Stroke Association (AHA/ASA) recommendations for treating acute ischemic stroke, the presence of acute ischemic features on non-contrast CT scans should not prevent the administration of alteplase. Based on the Third International Stroke Trial (IST-3) findings, the ASPECTS scoring system should not be used as a sole exclusion criterion for intravenous thrombolysis [20].

ASPECT score and mechanical thrombectomy

Mechanical thrombectomy is an endovascular recanalization of arteries, typically performed in proximal artery occlusion (middle cerebral artery, basilar artery, and carotid terminal). The ASPECT score of 6 becomes one of the criteria for mechanical thrombectomy. According to the 2016 HERMES (Highly Effective Reperfusion Evaluated in Multiple Endovascular Stroke) meta-analysis, lower ASPECT score (S) values are associated with poor therapeutic outcomes. When analyzing ischemic stroke imaging, the extent of the infarct lesion is regarded as a predictor of reperfusion therapy outcome. The majority of studies excluded patients with an ASPECTS score of 5, making it impossible to determine the efficacy of mechanical thrombectomy in this population. The ability of the ASPECTS scoring system to predict the outcome of mechanical thrombectomy has been a topic of debate due to contradictory findings between studies. According to Bhatt et al., a low ASPECT score (0 to 5) was related to bleeding transformation in mechanical thrombectomy [20]. However, Mourand et al. reported that mechanical thrombectomy in patients with low ASPECTS values (ranging from 0 to 5) yielded favorable outcomes without increasing the risk of hemorrhagic transformation [21].

The ASPECT scoring system is a straightforward system applied via CT scan or MRI. This system is more sensitive when performed with the DWI-MRI modality, particularly within three hours of the onset of symptoms. The ASPECTS scoring system is no longer used to determine intravenous thrombolysis eligibility. Nevertheless, according to the most recent recommendations, scoring remains one of the criteria for mechanical thrombectomy (ASPECTS 6) [20].

MRI or CT scan?

Even though the duration of an MRI scan is slightly longer than a CT scan, MRI-based selection for acute ischemic stroke patients can be completed in a similar amount of time as CT-based selection without delaying treatment or compromising the functional outcome. The acute stroke concept based on MRI is appropriate, expedient, and appears beneficial. The time-dependent quality indicators were superior to all stroke units and the comprehensive regional stroke units. Based on the MRI concept, it would be possible to observe high rates of recanalization procedures and fewer transient ischemic attack (TIA) diagnoses [22,23].

MRI and CT scans provide similar information. DWI/PWI and Perfusion CT revealed that the infarct core and ischemic penumbra were equivalents. Despite the similarity of their results, CT and MRI have distinct advantages and disadvantages that must be considered in the context of acute stroke. MRI is not accessible in all hospitals. Depending on the setting, performing an MRI for a stroke can be challenging without losing too much time before treatment is initiated. The primary benefits of MRI are direct visualization of the entire infarct area seen on DWI and whole-brain coverage, which permits the detection of small but clinically significant areas of hypo-perfusion. With time-of-flight magnetic resonance angiography (TOF-MRA), the Willis circle can be visualized in three minutes. There is no need for additional X-ray doses of iodinated contrast agents, so no nephrotoxicity or relevant allergic reactions are anticipated. In addition, technicians must be trained to perform stroke MRI rapidly to establish an adequate workflow during the hyper-acute phase of an ischemic stroke. The debate over whether CT or MRI is superior for acute imaging strokes should not obscure the ultimate objective, which is to increase the availability and efficacy of thrombolytic therapy. From this perspective, CT and MRI should be considered equivalent tools, and whatever techniques are available at each institution should be utilized for the acute stroke patient's greatest benefit [3,4,15].

## Conclusions

Multimodal imaging provides useful information for diagnosing ischemic stroke, selecting appropriate patients for thrombolytic therapy, and predicting the prognosis of ischemic stroke. MRI can detect ischemic stroke within the first few hours of stroke onset. Relying solely on one or a few parameters may not be sufficient, but comprehensively aggregating information from each MRI sequence (i.e., DWI, FLAIR, GRE {T2*gradient recalled echo}, and PWI) and using multiple mismatch parameters (DWI-FLAIR mismatch and PWI-DWI mismatch) may be more helpful in establishing the indications for MRI-based thrombolysis.
